# Supplementary material for: Well-being as a function of person-country fit in human values
Source: Nat Commun. 2020 Oct 13;11:5150. doi: 10.1038/s41467-020-18831-9 (PMC7554046; doi:10.1038/s41467-020-18831-9)
Supplement: Supplementary file 1 — Supplementary Information [file 41467_2020_18831_MOESM1_ESM.pdf]

**Supplementary Information of**

# Well-being as a Function of Person-Country Fit in Human Values

**Hanel et al.**

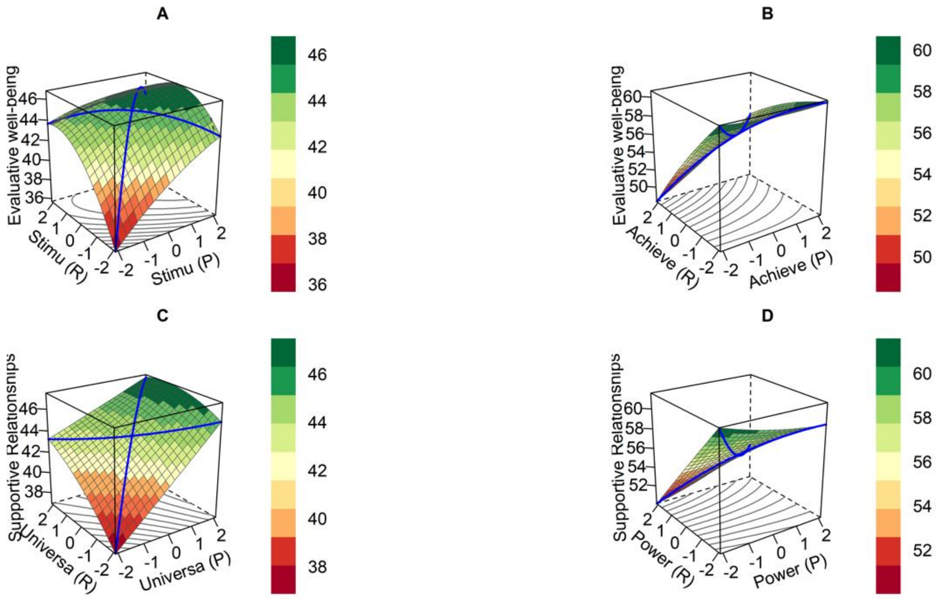


*Supplementary Figure 1*. Four response surface plots based on the multilevel polynomial regression results with individual (P) and regional (R) level values as predictors, including control variables. Predictors were standardized for illustrative purposes.

## Supplementary Methods

We provide here a step-by-step description of our analytical approach.

1) We T-transformed the six dependent variables (standard scores with *M* = 50 and *SD* = 10), because T-scores can be used as an effect size where a 2-point difference represents a small effect, a 5-point difference a medium effect, and an 8-point difference a large effect . Note that this does not change the pattern of correlation (*r*s = 1 between untransformed and transformed variables).
2) We mean-centered all 10 value types in line with guidelines for moderated regression analyses [1]. For example, we computed the mean of security and subtracted it from the security score of each participant.
3) We computed the country average for each of the ten value types, using the mean-centred value scores.
4) We performed 10 (value types) × 6 (well-being variables) × 2 (country vs regions) = 120 multi-level polynomial regression. Specifically, we entered the linear terms of individual value scores, the country (region) averages, the two respective quadratic terms which we obtained by multiplying separately each of the two linear terms with itself, and the interaction term as predictors. Additionally, we included the sample size of the country (region) as covariate and country (region) as random intercept. However, note that the pattern of result remained similar when we removed the control variable and the random effect (i.e., when we performed a standard polynomial regression).

## Supplementary Notes

Additionally, we tested whether person-environment value-fit differs across Eastern and Western European countries. While Schwartz and Bardi[2] report some value differences between Eastern and Western European countries, we expect that the rationale why value congruence should appear is valid even though value priorities may differ to a small extent between countries (also note that differences in value priorities between Eastern and Western European countries are decreasing[3]).

**Method.** The Eastern European countries we used in our analyses were Albania, Bulgaria, Czech Republic, Estonia, Hungary, Lithuania, Poland, Russia, Slovenia, Slovakia, Ukraine, and Kosovo ($N$ = 17,983, for individual-regional fit analysis, i.e., after excluding all regions with sample sizes < 100). The Western European countries were Belgium, Switzerland, Germany, Denmark, Spain, Finland, France, United Kingdom, Ireland, Italy, Netherlands, Norway, Portugal, and Sweden ($N$ = 23,100).

**Results.** We tested whether the pattern of results was different for Eastern and Western European countries. This was not the case. For example, the interaction terms of individual-regional fit for Eastern and Western European countries correlated across all 11×6-interaction terms with $r(64) = .61$, $p < .001$. Also, as Welsh's t-tests revealed, neither the raw numerical values of the interaction terms, $t(129.53) = 0.94$, $p = .35$, nor the absolute values differed from each other, $t(129.25) = 0.62$, $p = 54$.

## Supplementary Methods

To compute difference scores, we subtracted the country average from the individual response. We then correlated this difference score with the dependent variables. To compute the “absolute difference score”, we used the absolute value.

Supplementary Table 1

*Results for (absolute) difference score analyses*

| | | Difference score | | Absolute difference score | |
|---|---|---|---|---|---|
| DV | IV | *r* | *p* | *r* | *p* |
| Evaluative well-being | Security | .01 | .0092 | .01 | .1242 |
| | Tradition | .01 | .0025 | .01 | .0186 |
| | Conformity | .03 | 0 | .02 | .0009 |
| | Benevolence | .11 | 0 | -.1 | 0 |
| | Universalism | .07 | 0 | -.05 | 0 |
| | Self-direction | .14 | 0 | -.07 | 0 |
| | Stimulation | .12 | 0 | -.02 | 0 |
| | Hedonism | .15 | 0 | -.05 | 0 |
| | Achievement | .1 | 0 | 0 | .8779 |
| | Power | .04 | 0 | -.01 | .0016 |
| Emotional well-being | Security | .01 | .0698 | 0 | .8257 |
| | Tradition | -.01 | .0285 | .01 | .1059 |
| | Conformity | .02 | 0 | .01 | .2782 |
| | Benevolence | .1 | 0 | -.09 | 0 |
| | Universalism | .06 | 0 | -.05 | 0 |
| | Self-direction | .17 | 0 | -.09 | 0 |
| | Stimulation | .16 | 0 | -.04 | 0 |
| | Hedonism | .19 | 0 | -.07 | 0 |
| | Achievement | .11 | 0 | -.03 | 0 |
| | Power | .06 | 0 | -.01 | .0034 |
| Functioning | Security | .1 | 0 | -.02 | .0009 |
| | Tradition | .05 | 0 | .02 | 0 |
| | Conformity | .08 | 0 | .03 | 0 |
| | Benevolence | .25 | 0 | -.1 | 0 |
| | Universalism | .21 | 0 | -.06 | 0 |
| | Self-direction | .35 | 0 | -.1 | 0 |
| | Stimulation | .25 | 0 | -.03 | 0 |
| | Hedonism | .24 | 0 | -.05 | 0 |
| | Achievement | .25 | 0 | -.02 | .0002 |
| | Power | .12 | 0 | .03 | 0 |
| Vitality | Security | 0 | .358 | -.02 | .0007 |
| | Tradition | -.05 | 0 | 0 | .9532 |
| | Conformity | -.01 | .1116 | -.01 | .1471 |
| | Benevolence | .07 | 0 | -.07 | 0 |
| | Universalism | .05 | 0 | -.04 | 0 |

| | | | | | |
|---|---|---|---|---|---|
| | Self-direction | .16 | 0 | -.08 | 0 |
| | Stimulation | .17 | 0 | -.05 | 0 |
| | Hedonism | .17 | 0 | -.07 | 0 |
| | Achievement | .13 | 0 | -.04 | 0 |
| | Power | .07 | 0 | -.01 | .0149 |
| Community well-being | Security | 0 | .8575 | .02 | 0 |
| | Tradition | .06 | 0 | -.01 | .1931 |
| | Conformity | .04 | 0 | 0 | .3994 |
| | Benevolence | .11 | 0 | -.06 | 0 |
| | Universalism | .1 | 0 | -.04 | 0 |
| | Self-direction | .03 | 0 | -.04 | 0 |
| | Stimulation | .04 | 0 | -.02 | 0 |
| | Hedonism | .04 | 0 | -.05 | 0 |
| | Achievement | .02 | .0003 | -.01 | .0037 |
| | Power | -.01 | .0174 | -.04 | 0 |
| Supportive relationships | Security | .06 | 0 | -.01 | .0058 |
| | Tradition | .03 | 0 | .01 | .0298 |
| | Conformity | .03 | 0 | .03 | 0 |
| | Benevolence | .22 | 0 | -.13 | 0 |
| | Universalism | .15 | 0 | -.07 | 0 |
| | Self-direction | .17 | 0 | -.08 | 0 |
| | Stimulation | .12 | 0 | -.02 | .0001 |
| | Hedonism | .16 | 0 | -.05 | 0 |
| | Achievement | .12 | 0 | -.01 | .0418 |
| | Power | .04 | 0 | 0 | .5078 |

*Note*. 0: $p < .0001$. We do not interpret the correlations because difference scores cannot be interpreted [5].

## Supplementary References

1. Aiken, L. S. & West, S. G. *Multiple regression: Testing and interpreting interactions*. (Sage Publications, 1991).
2. Schwartz, S. H. & Bardi, A. Influences of adaptation to communist rule on value priorities in Eastern Europe. *Polit. Psychol.* **18**, 385–410 (1997).
3. Akaliyski, P. United in diversity? The convergence of cultural values among EU member states and candidates. *Eur. J. Polit. Res.* **58**, 388–411 (2018).
4. United Nations Developmental Programme. Human Developmental Report: Human Development Index (HDI). (2014).
5. Edwards, J. R. Alternatives to difference scores: Polynomial regression analysis and response surface methodology. in *Advances in measurement and data analysis* (eds. Drasgow, F. & Schmitt, N. W.) 350–400 (Jossey-Bass, 2002).
