# Supplementary material for: Well-being as a function of person-country fit in human values
Source: Nat Commun. 2020 Oct 13;11:5150. doi: 10.1038/s41467-020-18831-9 (PMC7554046; doi:10.1038/s41467-020-18831-9)
Supplement: Supplementary file 4 — Description of Additional Supplementary Files [file 41467_2020_18831_MOESM4_ESM.pdf]

Description of Additional Supplementary Files

File Name: Supplementary Data 1
Description: Detailed findings of individual-country-level fit: all coefficients of the polynomial regression and the response surface analysis

File Name: Supplementary Data 2
Description: Detailed findings of individual-region-level fit: all coefficients of the polynomial regression and the response surface analysis

File Name: Supplementary Data 3
Description: Detailed findings of individual-country-level fit: all coefficients of the polynomial regression and the response surface analysis (controlled for age and gender)

File Name: Supplementary Data 4
Description: Detailed findings of individual-region-level fit: all coefficients of the polynomial regression and the response surface analysis (controlled for age and gender)

File Name: Supplementary Data 5
Description: Multi-level polynomial regressions with controlling for HDI[4]

File Name: Supplementary Data 6
Description: Multi-level polynomial regressions controlling for individual-level age, gender, education level, income, country-level age and the Human Developmental Index”
